# Multi-oocyte follicles in the bovine ovary: occurrence, activation, and growth during early in vitro folliculogenesis in a dynamic culture bioreactor

**DOI:** 10.1007/s10815-025-03600-8

**Published:** 2025-08-09

**Authors:** Andrea Candela, Vincenza De Gregorio, Vincenzo Genovese, Angela Travaglione, Mario Cimmino, Riccardo Talevi, Roberto Gualtieri

**Affiliations:** 1 Department of Biology, University of Naples “Federico II,” Complesso Universitario Di Monte S. Angelo, Via Cinthia, 80126 Naples, Italy; 2 IVF Research, Education, Development S.R.L., Via Josemaria Escrivà, 68, 81100 Caserta, Italy

**Keywords:** Multi-oocyte follicles, Follicle growth, In vitro folliculogenesis, Dynamic culture, Perifusion bioreactor

## Abstract

**Purpose:**

Multi-oocyte follicles (MOFs) have been identified in various mammals, including humans, yet their origin and function remain controversial. This study aimed to investigate the occurrence and early developmental dynamics of MOFs in bovine ovaries, comparing them to single-oocyte follicles (SOFs).

**Methods:**

Ovarian tissues from *Bos taurus taurus* individuals, including one case with an unusually high MOF incidence, were cultured in vitro under dynamic conditions. MOF frequency and their progression through early folliculogenesis stages were assessed histologically and via confocal microscopy.

**Results:**

MOFs were observed at varying frequencies, with one individual showing a notably high incidence (15.8%). In vitro culture confirmed that MOFs can activate and progress to secondary stages, similar to SOFs. However, differences in activation rates and oocyte number dynamics were noted between MOFs and SOFs, and between fresh and cultured tissues. No de novo formation of MOFs was detected in vitro.

**Conclusions:**

MOFs retain the capacity for early folliculogenesis comparable to SOFs, without increased atresia. Their stable frequency postculture supports a prenatal origin. These findings offer new insights into MOF biology and suggest a possible physiological relevance in mammalian reproductive systems.

## Introduction

During female sex differentiation, oogonia undergo mitosis followed by incomplete cytokinesis giving rise to nests of germ cells connected through intercellular bridges. After initiation of meiosis and arrest at the prophase diplotene stage, during the second trimester in humans, and after birth in mice, germ-cell nest break down and flattened pregranulosa cells surround each oocyte to form primordial follicles [1, 2]. However, primordial follicles consisting of two or more oocytes enveloped by granulosa cells and a single follicle basal lamina, termed multiple-oocyte follicles (MOFs), have been reported in several mammals [3, 4]. Three main hypotheses have been proposed to explain the origin of MOFs. They could generate as consequence of (1) fusion of individual follicles during folliculogenesis due to ruptures of follicle basal lamina and the invasiveness of granulosa cells [5–7], (2) failure of germ-cell nest breakdown [8, 9], or (3) MOFs could represent a natural polymorphism, stemming from a range of potential numerical combinations of oocytes and pregranulosa cells [10]. The occurrence of a low number of MOFs is a common feature in humans [4], mice [9], rabbits [11], pigs [12], sheeps [13], and cattle [14], while in other mammals such as cotton rats [15] and dogs [16], MOFs can account up to the 40% of the ovarian follicle population. In *Bos taurus taurus* females, the prevalence of MOFs ranges from 0.3 to 5.4% [17]. Although MOFs have often been regarded as pathological entities, several reports in the human indicate that MOFs can successfully complete their growth and meiotic maturation giving rise to normal metaphase II oocytes. Mature oocytes enclosed in the same zona pellucida, termed conjoined oocytes, have been rarely retrieved by binovular MOFs in women undergoing assisted reproduction [18–20] and were capable of giving rise to live births [21–23]. In most cases, the size and maturation stage of oocytes in binovular follicles were asynchronous, though Magdi et al. (2020) reported a case of dizygotic twinning live births from two mature conjoined oocytes after intracytoplasmic sperm injection (ICSI) [24]. Far less is known on the dynamic of early folliculogenesis in MOFs compared to the population of SOFs. In the human, MOFs containing 2 or 3 oocytes were observed in 98% of 117 ovaries of women aged 18–52 years, and their frequency (0.06–2.44%) was not age-dependent [25]. Moreover, though MOFs were reported to undergo atresia before puberty, they were suggested to do so at rates similar to SOFs during the adult life-span [25–28]. In bitches, cows, and rabbits, MOFs have been observed in all stages of folliculogenesis, from primordial to peri-ovulatory follicles [11, 17]. The main aims of the present study were to determine the ability of primordial MOFs to activate and successfully progress to the primary and secondary follicle stages compared to the activation and growth dynamics of SOFs. We recently established a dynamic in vitro culture system for bovine and human ovarian tissue in a perifusion bioreactor (PB) that significantly improves follicle quality and viability, percentage and health of secondary follicles, overall tissue health, and steroid secretion compared to conventional static culture [29, 30]. Herein, we adopted such an in vitro culture system to study the incidence of MOFs and SOFs in fresh and cultured bovine ovarian cortical tissue and followed follicle activation, growth, and viability in tissues recovered from different individuals, including one subject with an unusually high occurrence of MOFs, which has not been previously observed in *Bos taurus taurus*. Our findings shed light on the dynamic and physiological function of MOFs during early folliculogenesis in the bovine animal model.

## Materials and methods

### Chemicals and consumables

Leibovitz’s L-15 medium, α-MEM GlutaMAX medium, insulin transferrin selenium (ITS) 100 ×, Live/dead Fixable Far-Red Stain were purchased from Invitrogen (Milan, Italy). Penicillin streptomycin 100 ×, amphotericin B 250 μg/mL, bovine serum albumin (BSA), L-ascorbic acid, L-glutamine 200 mM, propidium iodide, methanol, benzyl alcohol/benzyl benzoate (BABB), periodic acid Shiff (PAS), and eosin-Y were purchased from Sigma-Aldrich (Milan, Italy). Mayer’s haematoxylin and paraffin wax were from Carlo Erba (Milan, Italy). The tissue chopper was purchased from McIlwain, Mickle Laboratory Engineering Company Ltd. (Surrey, UK). Pump tubing was purchased from Cole-Parmer (Milan, Italy) and the peristaltic pump from Ismatec (Enco, Venice, Italy).

### Collection and preparation of ovarian tissue and dynamic culture in PB

Bovine ovaries collected at the time of slaughter (Slaughterhouse Di Tella, San Marcellino, Caserta, Italy; CEE accreditation number 1403/M) from 4 individuals (*n* = 4; age 8–24 months) were transported within 2 h to the lab in Leibovitz’s L-15 supplemented with 1% penicillin streptomycin (P/S) and 1 μg/mL amphotericin-B at 4 °C. Ovaries were cut into cortical slices (~ 0.6-mm thick) using a tissue slicer and further dissected in cortical strips 1 mm × 1 mm × 0.5 mm through the use of a tissue chopper (McIlwain, Mickle Laboratory Engineering Company, Ltd., Surrey, UK). After two washes in handling medium, groups of 10 ovarian cortical strips from each individual were randomly distributed into a sterile perifusion bioreactor (PB) for dynamic culture at a flow rate of 4 mL/min as previously reported [29, 30]. The cortical strips were cultured for 14 days, replacing half the volume with fresh medium every 2 days. At the end of the dynamic culture, 6 cortical strips from each PB were collected and processed for histology and 4 strips for viability assessments. Fresh uncultured strips were processed for histology and viability assessment as day 0 controls.

### Histology

Cortical strips fixed in Bouin’s and embedded in paraffin were cut into 5-μm-thick serial sections and stained with haematoxylin and eosin or with periodic acid Schiff (PAS). Follicle staging was evaluated by two blind expert observers and scored according to Gougeon’s criteria [31] as follows: primordial follicles have a single layer of flat GCs, primary follicles have a complete single layer of cuboidal GCs, and secondary follicles have two or more complete layers of cuboidal GCs. For PAS staining, sections were treated with periodic acid solution for 10 min at room temperature (RT), rinsed in distilled water, and immersed in Schiff’s reagent for 35 min in the dark. Slides were washed in sulfuruos acid solution (NaSO_3_ 10% w/v in 0.05 M HCl), rinsed for 5 min in tap water, counterstained in haematoxylin solution for 50 s, rinsed, dehydrated, cleared, and mounted in xylene-based mounting media.

### Viability assessment

Strips were incubated, under shaking, for 3 h at 4 °C, in Dulbecco’s PBS (phosphate buffered saline) with 1 μg/mL Live/Dead Fixable Far-Red Stain, washed in fresh dPBS for 30 min, fixed in 4% paraformaldehyde for 2 h at RT, washed in fresh PBS, and incubated at 4 °C overnight in a permeabilization solution consisting of triton 2% in PBS supplemented with propidium iodide 10 μg/mL. The live/dead probe is resistant to fixation and reacts with free amines and is excluded by cells with intact membranes. Strips were then optically cleared using BABB clearing protocol [32]. Briefly, samples were serially incubated in solutions of 50%, 70%, 80%, 90%, 100%, and 100% methanol, each for 30 min subsequently in a 1:1 solution composed of methanol and BABB for 4 h, and finally in absolute BABB for 24 h. To avoid compression, strips were mounted in BABB solution on a glass slide with 3 spacer coverslips (0.17 mm) placed on each side of the samples and covered with a coverslip. Analysis was carried out with a Leica TCS SP5 confocal scanning laser microscope (Leica Microsystems, Wetzlar, Germany) using a 496-nm Argon laser for visualizing the nuclear label (propidium iodide) and a 633-nm helium–neon laser for the live/dead probe. Each strip was traversed using the *z*-position control, and fields to a depth of 300 μm from the tissue surface were imaged using a 63 × glycerol immersion objective.

### Statistical analysis

For each experiment, data were reported as cumulative percentages. Statistical analysis was performed by Fisher’s exact test for pairwise comparisons when overall significance was detected.

## Results

**Fig. 1:**
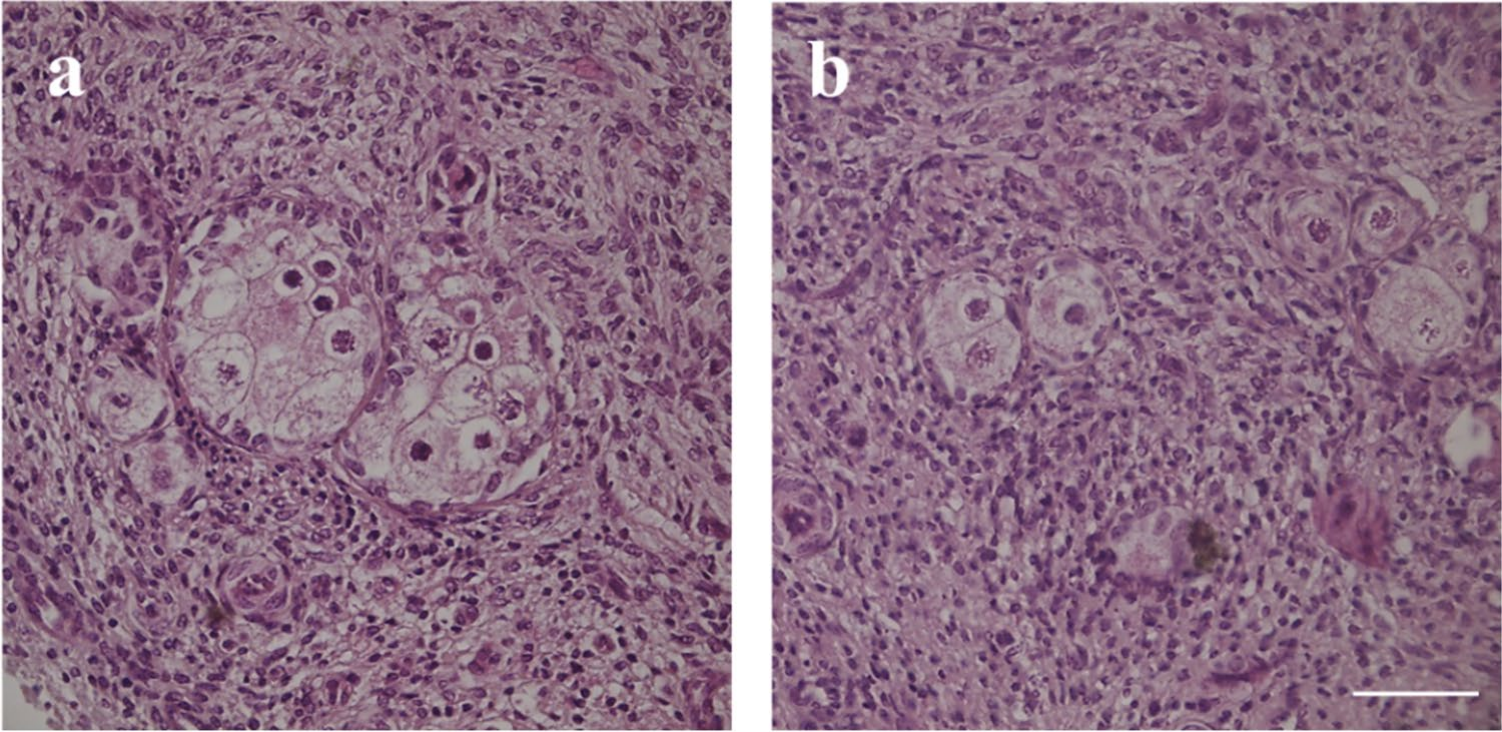
Representative images of hMI (**a**) and 1MI (**b**) in fresh tissue. Bar = 50 µm

Histological analysis of fresh and cultured ovarian tissues of the four individuals analysed showed the presence of MOFs in all samples. In our experience on early folliculogenesis in vitro, MOFs are usually retrieved at a low frequency in bovine ovaries. One individual had a markedly high frequency of MOFs and has been henceforth termed as high MOF individual (hMI), compared to the remaining three individuals, termed low MOF individuals (lMI). A total number of 656 follicles was scored in fresh tissue of hMI, and 15.8% of the total follicle population was accounted by MOFs containing 2 to 10 oocytes (Table 1, Fig. 1a). A total number of 1162 follicles were scored in the three lMI fresh tissues, and MOFs, containing 2 to 3 oocytes accounted for the 2.7% of the total follicle population (Table 1, Fig. 1b).

**Table 1:** Histological characterization of MOFs in hMI and lMI: MOF numbers, stages, and numbers of oocytes enclosed in follicles

	MOFs/total follicles	Follicle stage	No. of oocyte per follicle								
			2	3	4	5	6	7	8	9	10
hMI											
Fresh tissue	104/656	Primordial	60	19	7	3	-	-	-	-	-
		Primary	2	5	1	-	4	1	1	-	1
		Secondary	-	-	-	-	-	-	-	-	-
Postculture	82/485	Primordial	18	7	1	1	-	-	-	-	-
		Primary	26	13	3	1	-	-	-	-	-
		Secondary	8	4	-	-	-	-	-	-	-
IMI											
Fresh tissue	32/1162	Primordial	22	3	-	-	-	-	-	-	-
		Primary	7	-	-	-	-	-	-	-	-
		Secondary	-	-	-	-	-	-	-	-	-
Postculture	15/638	Primordial	4	3	-	-	-	-	-	-	-
		Primary	7	1	-	-	-	-	-	-	-
		Secondary	-	-	-	-	-	-	-	-	-

**Fig. 2:**
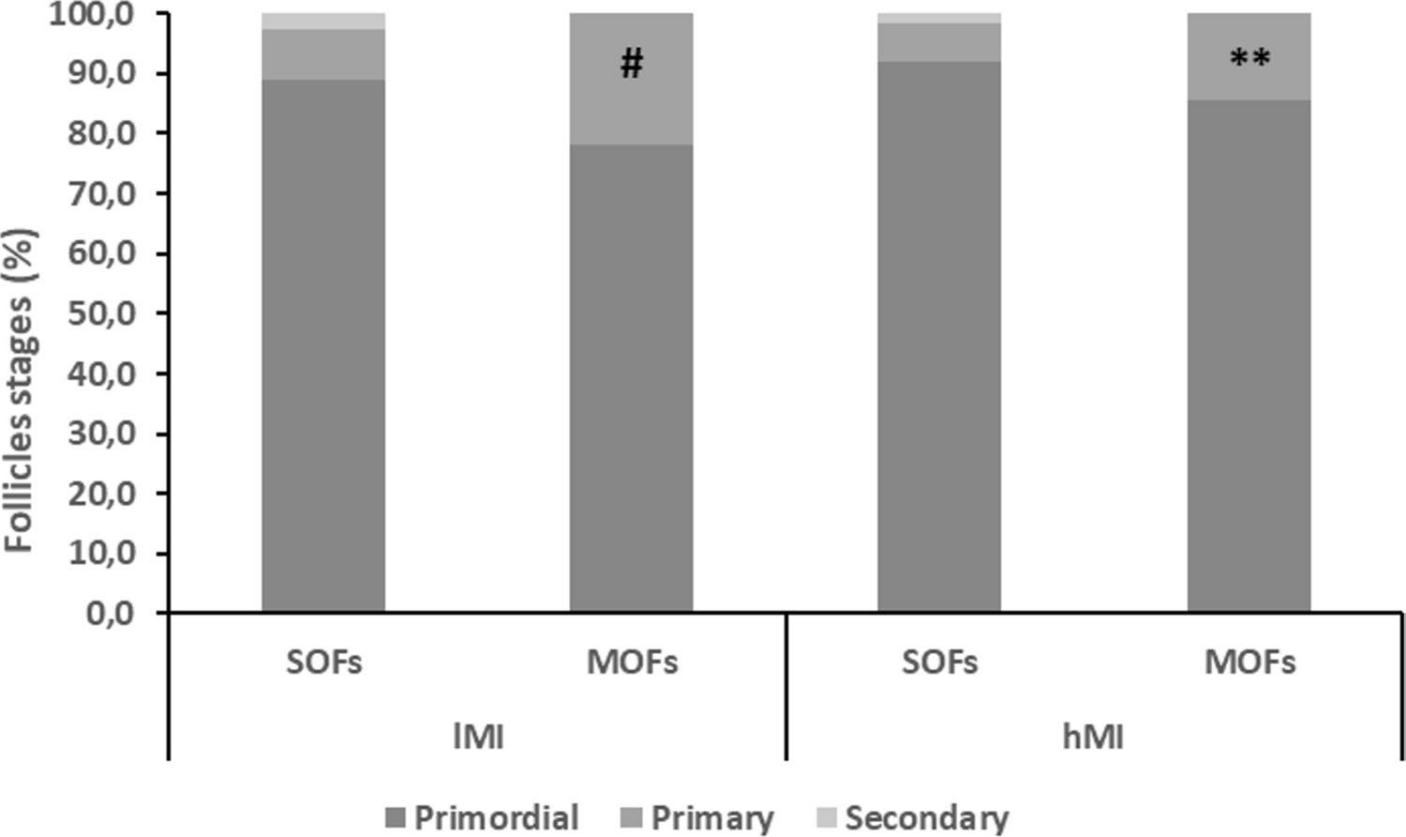
Follicle stages in fresh tissue of 1MI and hMI. #, *P* < 00.05 versus primary SOFs of 1MI; **, *P* < 00.01 versus primary SOFs of hMI

Histological analysis of fresh tissue samples demonstrated a similar high proportion of MOFs and SOFs at the primordial stage in hMI and lMI ovarian tissues (ns). However, the percentages of primary MOFs significantly exceeded those of primary SOFs in both lMI and hMI (Fig. 2).

In fresh hMI tissue, primordial MOFs (*n* = 89) contained 2–5 oocytes (Table 1). The percentages of primordial MOFs enclosing 2 to 5 oocytes were: 2, 67.4%; 3, 21.3%; 4, 7.9%; 5, 3.4%; mean primordial MOF oocyte number, 2.47. Primary MOFs (*n* = 15) contained 2 to 10 oocytes (Table 1), and the oocyte number per follicle was significantly higher compared to primordial follicles (*P* < 0.05). Percentages of primary MOFs enclosing 2 to 10 oocytes were: 2, 13.3%; 3, 33.3%; 4, 6.7%; 5, 0%; 6, 26.6%; 7, 6.7%; 8, 6.7%; 9, 0%; 10, 6.7%; mean primary MOF oocyte number, 4.80. Secondary MOFs were absent in fresh hMI tissue.

In lMI tissues, primordial MOFs (*n* = 25) contained 2–3 oocytes (Table 1; percentages of primordial MOFs enclosing 2 to 3 oocytes: 2, 88%; 3, 12%), and the mean primordial MOF oocyte number was 2.12. Primary MOFs (*n* = 7) enclosed 2 oocytes, and the mean primary MOF oocyte number was 2. Secondary MOFs were absent in fresh lMI tissues.

**Fig. 3:**
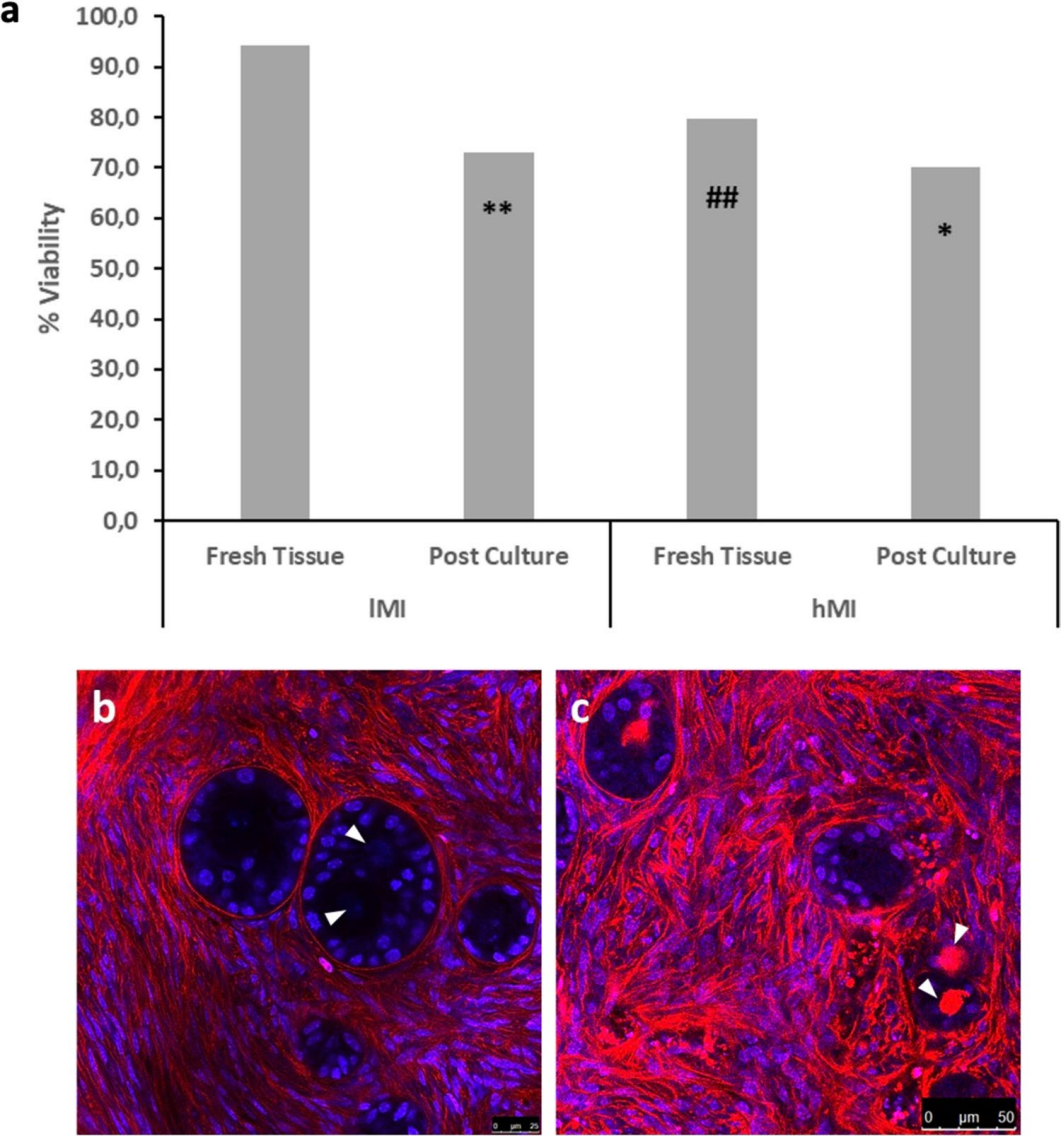
Viability assessment. **a** Percentages of viable follicles before and after 14 days of dynamic culture in 1MI and hMI. *, *P* < 00.05 and **, *P* < 00.01 versus corresponding fresh tissue; ##, *P* < 00.01 versus 1MI|fresh tissue. **b**, **c** Representative confocal images of live (**b**) and dead (**c**) SOFs and MOFs. Blue, propidium iodide-stained nuclei; red, extracellular matrix, basal lamina, and dead oocytes stained by live-dead far-red; white arrowheads, germinal vesicles of two live (**b**) or dead (**c**) oocytes in MOFs. Bar = 25 µm (**b**). Bar = 50 µm (**c**)

Confocal analysis of live-dead staining showed that 94.3% and 79.7% (*P* < 00.01) of follicles were viable in lMI and hMI tissues, respectively (Fig. 3a). Percentages of viable MOFs and SOFs were not significantly different in both hMI and lMI tissue samples as shown by representative confocal images (Fig. 3b, c).

**Fig. 4:**
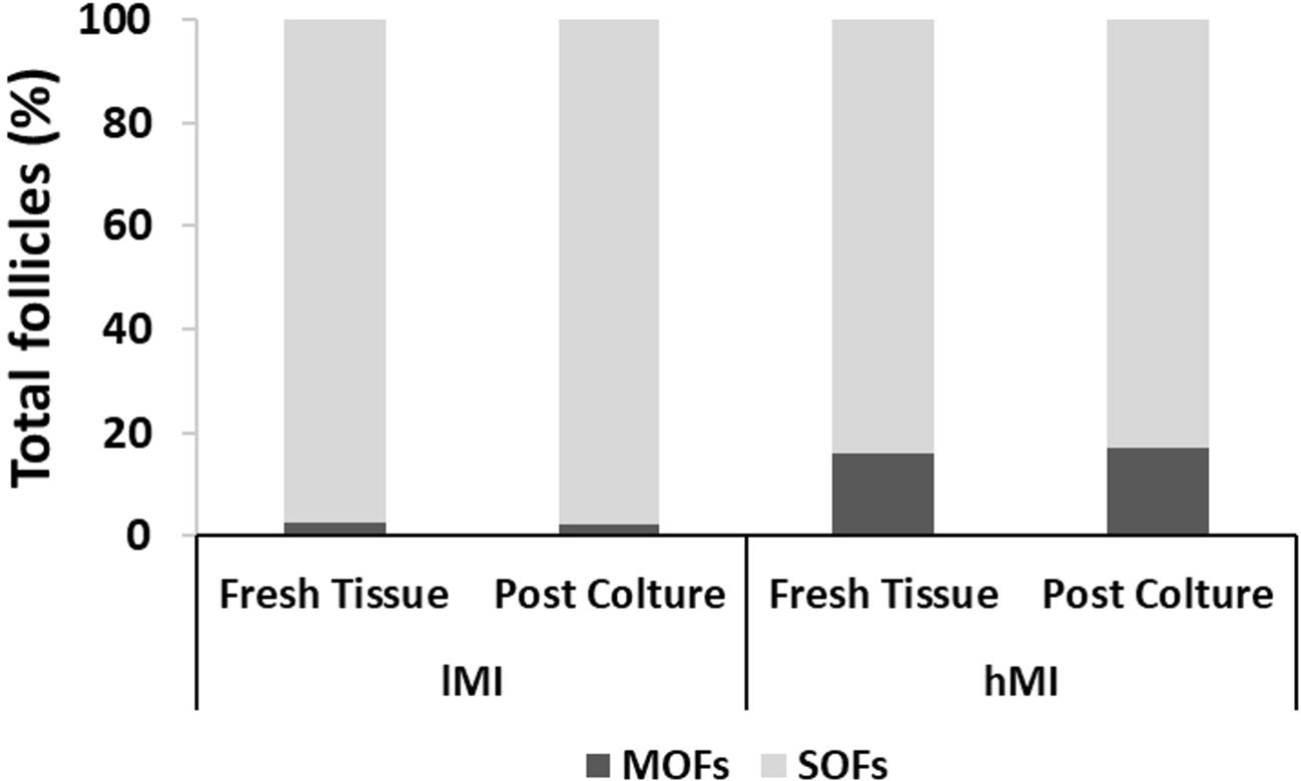
SOFs and MOF percentages before and after culture in 1MI and hMI

Ovarian cortical strips were analysed after 14 days of dynamic culture in PB. The percentage of MOFs in lMI and hMI remained constant before and after culture (Fig. 4), suggesting that 1) no fusions among follicles occurred during activation and growth in vitro, and 2) MOFs did not have an increased tendency to undergo atresia compared to SOFs.

**Fig. 5:**
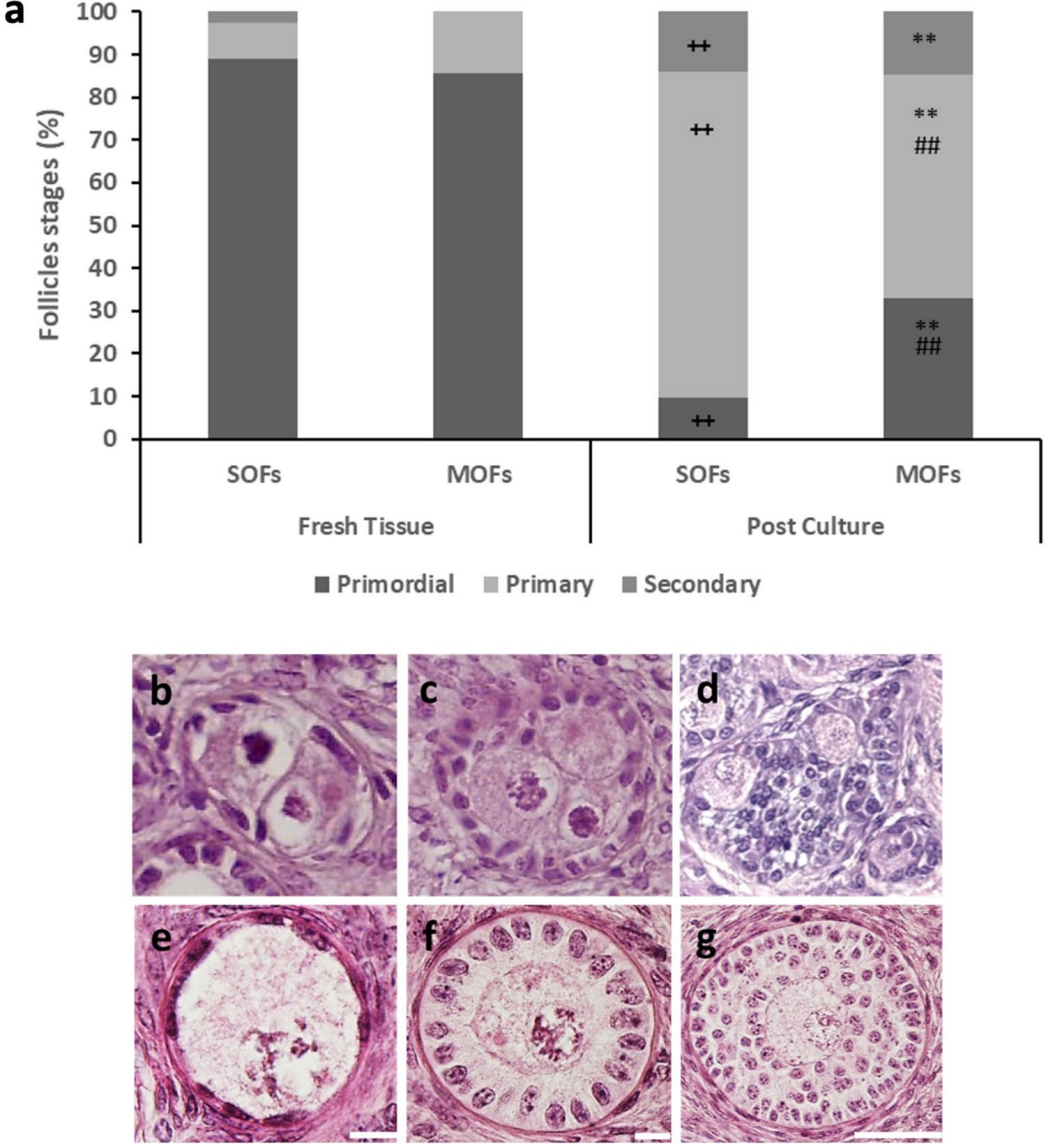
**a** Follicle stages in hMI before and after culture. **, *P* < 00.1 versus corresponding MOF stages in fresh tissue; +  +, *P* < 00.01 versus corresponding SOF stages in fresh tissue; ##, *P* < 00.01 versus corresponding SOF stages after culture. **b**–**g** Representative images of primordial (**b**, **e**), primary (**c**, **f**), and secondary (**d**, **g**) MOF and SOF, respectively. Bar = 10 µm (**b**, **c**, **e**, **f**). Bar = 50 µm (**d**, **g**)

In hMI cultured tissues, a significantly higher percentage of primordial and a decreased percentage of primary MOFs, compared to the proportions found in SOFs, were retrieved (Fig. 5a).

Histological analysis of follicle stages (Fig. 5b–d, MOFs; Fig. 5e–g, SOFs) in fresh and cultured hMI tissue showed that the proportion of primordial SOFs dropped from 89 to 10%, whereas the percentage of primordial MOFs dropped from 80 to 35% after culture. This indicates that, although both follicle types activate their growth during culture, a significantly lower proportion of primordial MOFs (56.2%) were able to do so compared to primordial SOFs (88.8%) over the 14-day culture period. Although the percentage of primary SOFs significantly exceeded that of primary MOFs, the proportions of secondary SOFs and MOFs were similar suggesting a similar competence of the two follicle populations to progress to the secondary stage during culture (Fig. 5a). The dynamic of activation and growth in SOFs of lMI was similar to that shown in hMI. The dynamics of activation and progression in MOFs of lMI were not shown given the very low numbers of MOFs retrieved.

In hMI cultured tissue, primordial MOFs (*n* = 27) contained 2–5 oocytes (percentages of primordial MOFs enclosing 2 to 5 oocytes: 2, 66.7%; 3, 25.9%; 4, 3.7%; 5, 3.7%; mean primordial MOF oocyte number, 2.44), primary MOFs (*n* = 43) had 2–5 oocytes (percentages of primary MOFs enclosing 2 to 5 oocytes:2, 60.5%; 3, 30.2%; 4, 7%; 5, 2.3%; mean primary MOF oocyte number, 2.51), secondary MOFs (*n* = 12) had 2–3 oocytes (percentages of secondary MOFs enclosing 2 or 3 oocytes: 2, 66.6%; 3, 33.4%; mean secondary MOF oocyte number, 2.33). Numbers of oocytes in MOFs of lMI were not shown given the very low numbers of MOFs retrieved.

**Fig. 6:**
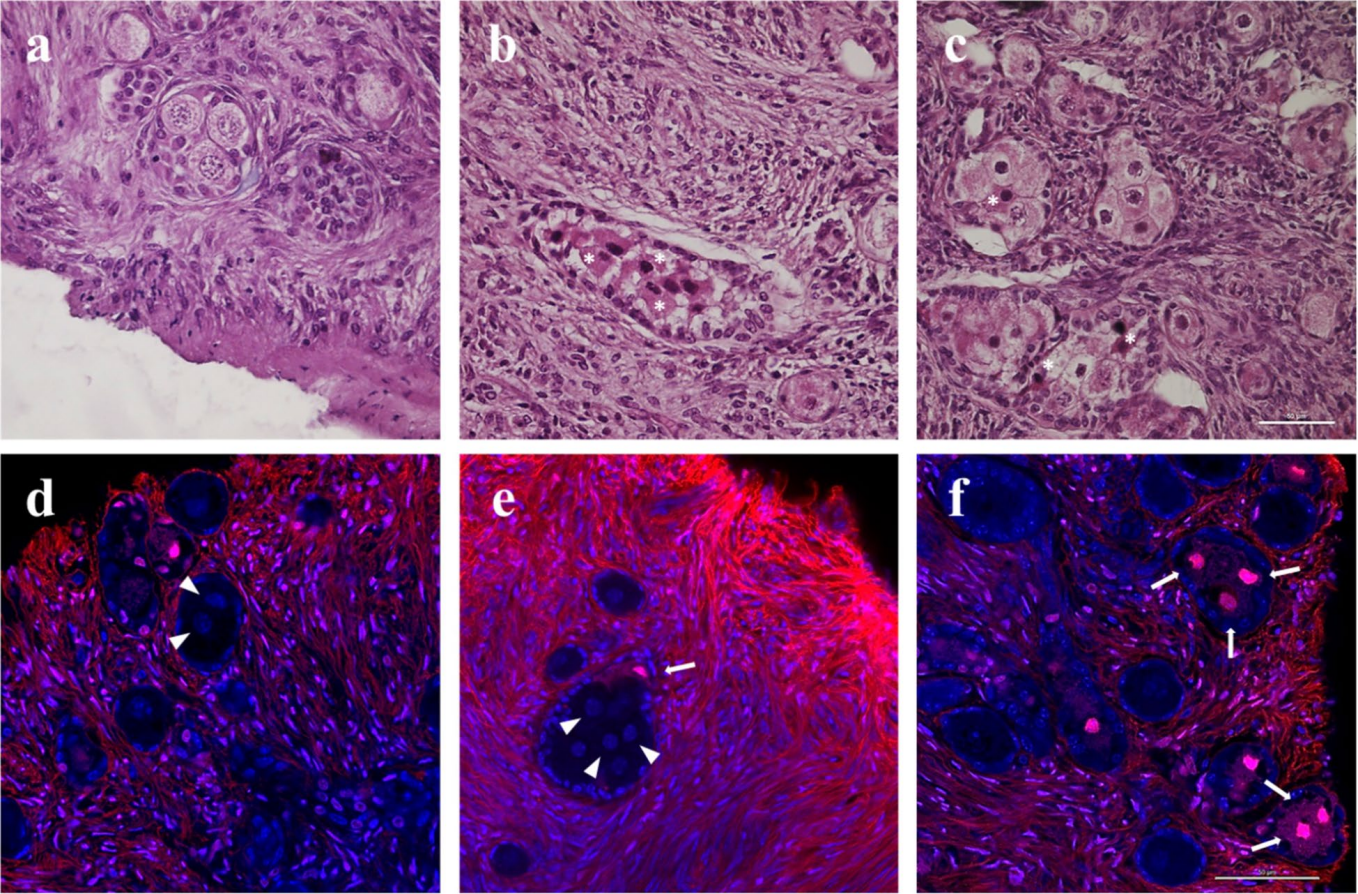
Representative histological and confocal images showing the health (**a**–**c**) and viability (**d**–**f**) of MOF oocytes in hMI. *, Eosinophilic, damaged oocytes; white arrowheads = germinal vesicles of live oocytes; white arrows, dead oocytes. Bar = 50 µm

Total follicle viability significantly decreased after culture in both lMI (*P* < 00.01) and hMI (*P* < 00.05) tissues (Fig. 3a). Loss of viability of one or more oocytes in hMI MOFs was occasionally observed after culture both in histological (Fig. 6a–c) and confocal (Fig. 6d–f) assessments.

**Fig. 7:**
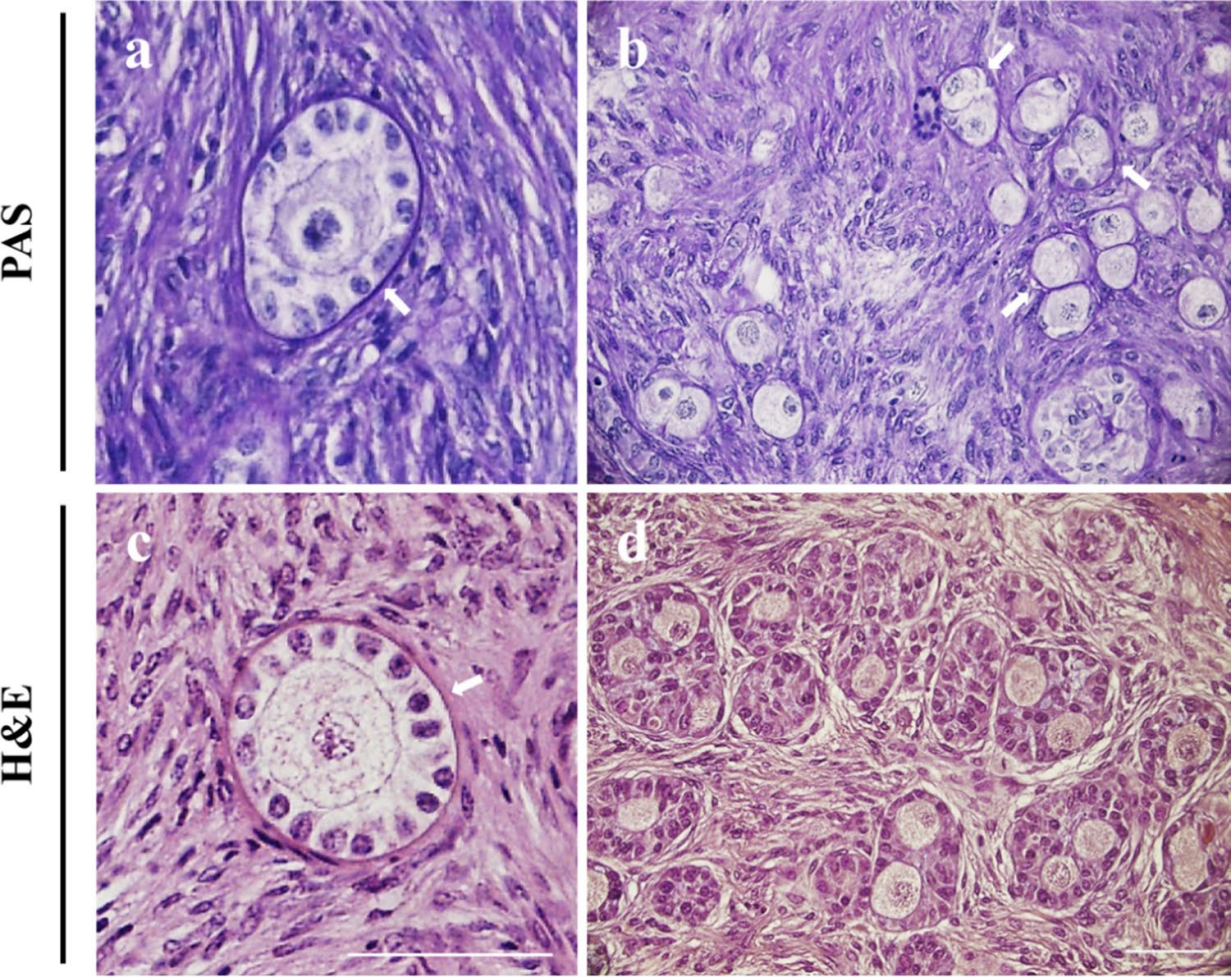
Representative images of SOFs and MOFs in hMI. White arrows, basal laminae. Bar = 50 µm

No figures of basal lamina breakdown or invasion of granulosa cells from one follicle to the neighboring follicles were detected in confocal optical sections (Fig. 6d–f) and histological sections (Fig. 7a–d), confirming that the number of MOFs is kept constant during culture.

## Discussion

During female sex differentiation, the germ-cell nest breakdown is a key early event in mammalian folliculogenesis, in which physiologically a single layer of pregranulosa cells surrounds each oocyte resulting in the formation of primordial follicles [1, 2]. However, the presence of MOFs in the ovary is relatively frequent in various species [4, 9, 11–16, 25], including *Bos taurus taurus* with an occurrence ranging from 0.3 to 5.4% of the total follicle population [17]. Our findings showed a high incidence of MOFs in one individual (15.8%) compared to the other three individuals in which MOF frequency is within the range reported in literature in this species (2.7%).

We are unable to provide a clear explanation for the unusually high occurrence of MOFs observed in this individual. Several studies across different species reported a higher frequency of MOFs in prepubertal subjects compared to postpubertal ones [12, 14, 33, 34]. Although we have previously analysed a considerable number of ovaries from *Bos taurus taurus* individuals aged 8 to 24 months—corresponding to pubertal or adult stages [29, 30, 35]—this represents, in our experience as well as to the best of our knowledge in the literature, the first documented case of such a high occurrence of MOFs in this species.

Several factors reportedly contribute to MOF origin, including genetic, chemical, and physical influences that may interfere with the breakdown of germ-cell nests. Foetal or neonatal exposure to environmental endocrine disruptors in various species [9, 35–38], along with altered gene expression in transgenic or knockout mice [6, 39, 40], and foetal irradiation in rats [41], have been reported to increase the frequency of MOFs. We cannot rule out the possibility that the hMI was exposed to unknown factors inducing this unusually high incidence of MOFs at the time of germinal nest breakdown.

Regardless of the underlying causes of the high frequency of MOFs in the hMI individual, this occurrence provided a valuable opportunity to assess their activation and growth in comparison with coexisting SOFs from the same individual.

The role and the functionality of MOFs in reproductive physiology remain a matter of debate. Several studies consider MOFs as abnormalities in terms of formation, activation, and progression through the stages of follicular development [5, 6, 39]. Conversely, although they occur at a very low frequency, MOFs could normally proceed through folliculogenesis as they have been observed from primordial to periovulatory stages in different species [4, 11, 17, 34]. In addition, the high incidence of MOFs in mouse lines selected for high fertility suggested a potential association between the MOF occurrence and increased reproductive capability [42]. A further direct evidence of MOF developmental competence is the retrieval of conjoined oocytes in ART patients, which have led to full-term births [20–24].

Herein, several findings differed between fresh and in vitro cultured tissue. In uncultured tissue of hMI and lMI, the proportions of primary MOFs significantly exceeded those of primary SOFs. This could suggest either that primordial MOFs have a higher activation ability, or that MOFs spend a longer time at the primary stage compared to their SOFs counterparts. In cultured tissues, there is a remarkable drop of primordial follicles and a concomitant increase of primary follicles as mechanical fragmentation of ovarian cortical tissue followed by in vitro culture is known to promote dormant follicle activation through the Hippo pathway disruption [43–45]. Although data showed that MOFs are capable to activate and progress through the early stages of folliculogenesis, the frequencies of primordial and primary MOFs were higher and lower compared to the corresponding SOF stages, respectively. This observation, contrary to the in vivo findings, may suggest a lower activation tendency of the MOFs following in vitro culture. Nevertheless, the presence of a comparable proportion of secondary MOFs and SOFs in the hMI individual indicates that MOFs retain a normal capacity for follicular progression. Given the low frequency of MOFs, the lack of secondary MOFs in lMI cultured tissues was not unexpected, as after dynamic culture, the yield of secondary follicles is usually limited to 15–20% of the total follicle population [29, 30]. This is consistent with studies suggesting that the rarity of MOFs at advanced follicular stages is primarily due to their already low incidence at the primordial stage [46].

A second aspect differing between the fresh and the cultured tissues was the number of oocytes in dormant versus growing MOFs. Specifically, only in fresh hMI tissue, the mean oocyte number in primary MOFs almost doubled the corresponding number in primordial MOFs (4.8 vs 2.4, *P* < 00.05). In agreement with other studies, this could suggest that primary MOFs could form postnatally due to the fusion of neighboring activated follicles, through the infiltrative ability of granulosa cells [5, 7]. However, we did not observe figures of granulosa cell infiltration or breakage of the follicle basal lamina in histological and confocal analysis. Conversely, in hMI cultured tissue, the mean MOF oocyte number was kept constant through the primordial, primary, and secondary stages (2.4, 2.5, 2.3). One of the main differences between the theory of prenatal MOFs formation as a result of “nest breakdown failure” and the postnatal formation according to the hypothesis of granulosa cells’ invasiveness concerns the number of MOFs found in the adult ovary. In fact, in the case of a prenatal formation, the MOF pool should remain unchanged or at most decrease in the adult ovary, whereas according to the postnatal hypothesis, a de novo formation of MOFs could occur. Our results in hMI and lMI demonstrate that the percentage of total MOFs remains unchanged after culture suggesting that de novo formation does not occur in vitro. We cannot rule out the possibility that unknown factors present only in the in vivo environment promote the postnatal formation of MOFs as a result of fusion of activated follicles.

The literature comparing the ability of MOFs to undergo folliculogenesis in vitro with that of SOFs is limited. Most studies have been focused on the formation, oocyte functionality, and morphological features of MOFs in vivo, rather than on a detailed functional comparison with SOFs in vitro. To our knowledge, this is the first study in which MOF development and viability were assessed, comparing them to SOFs within the same individual. Confocal analysis of follicle viability in fresh and cultured tissues indicates that MOFs did not have an increased tendency to undergo atresia compared to SOFs. However, MOFs containing at least one or more dead oocytes were observed in both histological and confocal images. The observation of apparently viable MOFs with one or a few dead oocytes could cause a progressive reduction of the oocyte number in more advanced stages of folliculogenesis [8, 10].

Several studies detected differences among the oocytes contained in advanced MOF stages in terms of size and meiotic condition [11]. We did not observe evident size differences among oocytes contained in MOFs from the primordial to the secondary stages. This could suggest that the reported asynchrony in growth and meiotic maturation among oocytes in single advanced MOF stages could arise later, during the advanced preantral or the antral phase of folliculogenesis.

In conclusion, the present findings indicate that MOFs do not substantially differ from SOFs in their ability to undergo early folliculogenesis in vitro. MOFs demonstrated the capacity to activate and progress through the primordial, primary, and secondary stages without evident signs of increased atresia or compromised viability. While certain differences emerged between fresh and cultured tissues—particularly regarding oocyte number dynamics and follicle activation potential—these did not appear to hinder MOF development. Importantly, the maintenance of a constant proportion of MOFs after culture supports the hypothesis that de novo MOF formation does not occur in vitro, reinforcing the idea of a predominantly prenatal origin. Further studies are needed to explore whether environmental or molecular factors might promote postnatal MOF formation in vivo and to determine the developmental competence of MOFs beyond the early stages. These findings offer new insight into the developmental plasticity of MOFs and support their potential relevance in reproductive physiology.

## Data Availability

The data presented in this study are available on request from the corresponding author.

## References

[1] Tingen C, Kim A, Woodruff TK. The primordial pool of follicles and nest breakdown in mammalian ovaries. Mol Hum Reprod. 2009;15(12):795–803. 10.1093/molehr/gap073. 19710243 10.1093/molehr/gap073 PMC2776475

[2] Pepling ME. Follicular assembly: mechanisms of action. Reproduction. 2012;143(2):139–49. 10.1530/REP-11-0299. 22065859 10.1530/REP-11-0299

[3] Chaves MS, Azevedo HC, Luz VB, Ferreira-Silva JC, Barros I, Paiva SR, de Olivera Melo E, de Melo Magalhães Padilha D, de Figueirêdo Freitas VJ, Bartolomeu CC, Lemos Oliveira MA. Occurrence, morphology, and morphometry of follicles containing multiple oocytes in FecGE mutant Santa Inês ewes. Anim Reprod Sci. 2021;226:106690. 10.1016/j.theriogenology.2019.06.033. 10.1016/j.anireprosci.2021.106690 33561808

[4] Silva-Santos KC, Seneda MM. Multioocyte follicles in adult mammalian ovaries. Anim Reprod. 2011;8(3):58–67.

[5] Gaytán F, Morales C, Manfredi-Lozano M, Tena-Sempere M. Generation of multi-oocyte follicles in the peripubertal rat ovary: link to the invasive capacity of granulosa cells? Fertil Steril. 2014;101(5):1467–76. 10.1016/j.fertnstert.2014.01.037. 24581577 10.1016/j.fertnstert.2014.01.037

[6] Perez-Sanz J, Arluzea J, Matorras R, Gonzalez-Santiago N, Bilbao J, Yeh N, et al. Increased number of multi-oocyte follicles (MOFs) in juvenile p27Kip1 mutant mice: potential role of granulosa cells. Hum Reprod. 2013;28(4):1023–30. 10.1093/humrep/des436. 23300200 10.1093/humrep/des436 PMC4439520

[7] Harrath AH, Alrezaki A, Mansour L, Alwasel SH, Palomba S. Food restriction during pregnancy and female offspring fertility: adverse effects of reprogrammed reproductive lifespan. J Ovarian Res. 2017. 10.1186/s13048-017-0372-x. 29282125 10.1186/s13048-017-0372-x PMC5745764

[8] Reynaud K, Halter S, Tahir Z, Thoumire S, Chebrout M, Chastant-Maillard S. Les follicules polyovocytaires [Polyovular follicles]. Gynecol Obstet Fertil. 2010;38(6):395–7. 10.1016/j.gyobfe.2010.04.008. 20576549 10.1016/j.gyobfe.2010.04.008

[9] Jefferson W, Newbold R, Padilla-Banks E, Pepling M. Neonatal Genistein treatment alters ovarian differentiation in the mouse: inhibition of oocyte nest breakdown and increased oocyte survival. Biol Reprod. 2006;74(1):161–8. 10.1095/biolreprod.105.045724. 16192398 10.1095/biolreprod.105.045724

[10] Telfer E, Gosden RG. A quantitative cytological study of polyovular follicles in mammalian ovaries with particular reference to the domestic bitch (*Canis familiaris*). Reproduction. 1987;81(1):137–47. 10.1530/jrf.0.0810137. 10.1530/jrf.0.0810137 3668945

[11] Al-Mufti W, Bomsel-Helmreich O, Christides JP. Oocyte size and intrafollicular position in polyovular follicles in rabbits. Reproduction. 1988;82(1):15–25. 10.1530/jrf.0.0820015. 10.1530/jrf.0.0820015 3339576

[12] Stankiewicz T, Błaszczyk B, Udała J. A study on the occurrence of polyovular follicles in porcine ovaries with particular reference to intrafollicular hormone concentrations, quality of oocytes and their in vitro fertilization. Anat Histol Embryol. 2009;38(3):233–9. 10.1111/j.1439-0264.2009.00929.x. 19469770 10.1111/j.1439-0264.2009.00929.x

[13] Oliveira RL, Silva CB, Silva EO, Gerez JR, Santos MM, Sarapião FD, et al. Proliferative activity of multi-oocyte follicles in sheep ovaries. Small Ruminant Res. 2017;146:58–60. 10.1016/j.smallrumres.2016.12.004.

[14] Silva-Santos KC, Santos GMG, Siloto LS, Hertel MF, Andrade ER, Rubin MIB, et al. Estimate of the population of preantral follicles in the ovaries of *Bos taurus indicus* and *Bos taurus taurus* cattle. Theriogenology. 2011;76(6):1051–7. 10.1016/j.theriogenology.2011.05.008. 21722949 10.1016/j.theriogenology.2011.05.008

[15] Islam MR, Ichii O, Nakamura T, Irie T, Masum MA, Hosotani M, et al. Unique morphological characteristics in the ovary of cotton rat (*Sigmodon hispidus*). J Reprod Dev. 2020;66(6):529–38. 10.1262/jrd.2020-061. 32879182 10.1262/jrd.2020-061 PMC7768171

[16] Reynaud K, de Lesegno CV, Chebrout M, Thoumire S, Chastant-Maillard S. Follicle population, cumulus mucification, and oocyte chromatin configuration during the periovulatory period in the female dog. Theriogenology. 2009;72(8):1120–31. 10.1016/j.theriogenology.2009.07.006. 19775739 10.1016/j.theriogenology.2009.07.006

[17] Ireland JLH, Scheetz D, Jimenez-Krassel F, Themmen APN, Ward F, Lonergan P, et al. Antral follicle count reliably predicts number of morphologically healthy oocytes and follicles in ovaries of young adult cattle. Biol Reprod. 2008;79(6):1219–25. 10.1095/biolreprod.108.071670. 18768912 10.1095/biolreprod.108.071670

[18] Zeilmaker GH, Alberda AT, van Gent I. Fertilization and cleavage of oocytes from a binovular human ovarian follicle: a possible cause of dizygotic twinning and chimerism. Fertil Steril. 1983;40(6):841–3. 10.1016/S0015-0282(16)47490-X. 6653803 10.1016/s0015-0282(16)47490-x

[19] Ron-El R, Nachum H, Golan A, Herman A, Yigal S, Caspi E. Binovular human ovarian follicles associated with in vitro fertilization: incidence and outcome. Fertil Steril. 1990;54(5):869–72. 10.1016/S0015-0282(16)53948-X. 2226920 10.1016/s0015-0282(16)53948-x

[20] Coban O, Serdarogullari M, Pervaiz R, Soykok A, Bankeroglu H. Fertilization and development of oocytes with separated and conjoined zona pellucida recovered from polyovular follicles: description of two cases and a literature review. Zygote. 2021;29(4):282–5. 10.1017/S0967199420000878. 33468269 10.1017/S0967199420000878

[21] Cummins L, Koch J, Kilani S. Live birth resulting from a conjoined oocyte confirmed as euploid using array CGH: a case report. Reprod Biomed Online. 2016;32(1):62–5. 10.1016/j.rbmo.2015.09.012. 26602945 10.1016/j.rbmo.2015.09.012

[22] Yano K, Hashida N, Kubo T, Ohashi I, Koizumi A, Kageura R, et al. Repeated collection of conjoined oocytes from a patient with polycystic ovary syndrome, resulting in one successful live birth from frozen thawed blastocyst transfer: a case report. J Assist Reprod Genet. 2017;34(11):1547–52. 10.1007/s10815-017-1012-5. 28780721 10.1007/s10815-017-1012-5 PMC5699992

[23] Fu L, Chen S, Wang M, Huang G, Wang F, Lan Y, et al. Live birth from a blastocyst derived from a conjoined oocyte in a frozen embryo transfer cycle: a case report and a literature review. J Assist Reprod Genet. 2022;39(6):1351–7. 10.1007/s10815-022-02465-5. 35320445 10.1007/s10815-022-02465-5 PMC9174371

[24] Magdi Y. Dizygotic twin from conjoined oocytes: a case report. J Assist Reprod Genet. 2020;37(6):1367–70. 10.1007/s10815-020-01772-z. 32285296 10.1007/s10815-020-01772-z PMC7311585

[25] Gougeon A. Frequent occurrence of multiovular follicles and multinuclear oocytes in the adult human ovary. Fertil Steril. 1981;35(4):417–22. 10.1016/S0015-0282(16)45436-1. 7215567 10.1016/s0015-0282(16)45436-1

[26] Hartman CG. Polynuclear ova and polyovular follicles in the opossum and other mammals, with special reference to the problem of fecundity. Am J Anat. 1926;37(1):1–51. 10.1002/aja.1000370102.

[27] Bacsich P. Multinuclear ova and multiovular follicles in the young human ovary and their probable endocrinological significance. J Endocrinol. 1949;6(1):i. 18236545

[28] Dawson AB. Histogenetic interrelationships of oocytes and follicle cells; a possible explanation of the mode of origin of certain polyovular follicles in the immature rat. Anat Rec. 1951;110(2):181–97. 10.1002/ar.1091100206. 14857342 10.1002/ar.1091100206

[29] Barbato V, Genovese V, De Gregorio V, Di Nardo M, Travaglione A, De Napoli L, et al. Dynamic in vitro culture of bovine and human ovarian tissue enhances follicle progression and health. Sci Rep. 2023. 10.1038/s41598-023-37086-0. 37479791 10.1038/s41598-023-37086-0 PMC10361967

[30] Fragomeni G, De Napoli L, De Gregorio V, Genovese V, Barbato V, Serratore G, et al. Enhanced solute transport and steady mechanical stimulation in a novel dynamic perifusion bioreactor increase the efficiency of the in vitro culture of ovarian cortical tissue strips. Front Bioeng Biotechnol. 2024. 10.3389/fbioe.2024.1310696. 38390358 10.3389/fbioe.2024.1310696 PMC10882273

[31] Gougeon A. Regulation of ovarian follicular development in primates: facts and hypotheses. Endocr Rev. 1996;17(2):121–55. 10.1210/edrv-17-2-121. 8706629 10.1210/edrv-17-2-121

[32] Mazio C, Mavaro I, Palladino A, Casale C, Urciuolo F, Banfi A, et al. Rapid innervation and physiological epidermal regeneration by bioengineered dermis implanted in mouse. Mater Today Bio. 2024;25: 100949. 10.1016/j.mtbio.2024.100949. 38298559 10.1016/j.mtbio.2024.100949 PMC10827562

[33] Lucci CM, Amorim CA, Rodrigues AP, Figueiredo JR, Báo SN, Silva JR, et al. Study of preantral follicle population in situ and after mechanical isolation from caprine ovaries at different reproductive stages. Anim Reprod Sci. 1999;56(3–4):223–36. 10.1016/S0378-4320(99)00045-7. 10497918 10.1016/s0378-4320(99)00045-7

[34] Payan-Carreira R, Pires MA. Multioocyte follicles in domestic dogs: a survey of frequency of occurrence. Theriogenology. 2008;69(8):977–82. 10.1016/j.theriogenology.2008.01.013. 18358525 10.1016/j.theriogenology.2008.01.013

[35] Takashima-Sasaki K, Komiyama M, Adachi T, Sakurai K, Kato H, Iguchi T, et al. Effect of exposure to high isoflavone-containing diets on prenatal and postnatal offspring mice. Biosci Biotechnol Biochem. 2006;70(12):2874–82. 10.1271/bbb.60278. 17151444 10.1271/bbb.60278

[36] Iguchi T, Fukazawa Y, Uesugi Y, Takasugi N. Polyovular follicles in mouse ovaries exposed neonatally to diethylstilbestrol in vivo and in vitro. Biol Reprod. 1990;43(3):478–84. 10.1095/biolreprod43.3.478. 2271729 10.1095/biolreprod43.3.478

[37] Rodríguez HA, Santambrosio N, Santamaría CG, Muñoz-de-Toro M, Luque EH. Neonatal exposure to bisphenol A reduces the pool of primordial follicles in the rat ovary. Reprod Toxicol. 2010;30(4):550–7. 10.1016/j.reprotox.2010.07.008. 20692330 10.1016/j.reprotox.2010.07.008

[38] Susiarjo M, Hassold TJ, Freeman E, Hunt PA. Bisphenol A exposure in utero disrupts early oogenesis in the mouse. PLoS Genet. 2007;3(1): e5. 10.1371/journal.pgen.0030005. 17222059 10.1371/journal.pgen.0030005 PMC1781485

[39] Su W, Guan X, Zhang D, Sun M, Yang L, Yi F, et al. Occurrence of multi-oocyte follicles in aquaporin 8-deficient mice. Reprod Biol Endocrinol. 2013;11: 88. 10.1186/1477-7827-11-88. 24020646 10.1186/1477-7827-11-88 PMC3847684

[40] Yan C, Wang P, Demayo J, Demayo FJ, Elvin JA, Carino C, et al. Synergistic roles of bone morphogenetic protein 15 and growth differentiation factor 9 in ovarian function. Mol Endocrinol. 2001;15(6):854–66. 10.1210/mend.15.6.0662. 11376106 10.1210/mend.15.6.0662

[41] Mazaud Guittot SV, Guigon CJ, Coudouel NL, Magre S. Consequences of fetal irradiation on follicle histogenesis and early follicle development in rat ovaries. Biol Reprod. 2006;75(5):749–59. 10.1095/biolreprod.105.050633. 16855212 10.1095/biolreprod.105.050633

[42] Alm H, Kuhlmann S, Langhammer M, Tuchscherer A, Torner H, Reinsch N. Occurrence of polyovular follicles in mouse lines selected for high fecundity. J Reprod Dev. 2010;56(4):449–53. 10.1262/jrd.09-224H. 20519833 10.1262/jrd.09-224h

[43] Cordeiro CN, Christianson MS, Selter JH, Segars JH. In vitro activation: a possible new frontier for treatment of primary ovarian insufficiency. Reprod Sci. 2016;23(4):429–38. 10.1177/1933719115625842. 26787101 10.1177/1933719115625842

[44] Fabregues F, Ferreri J, Calafell JM, Moreno V, Borrás A, Manau D, et al. Pregnancy after drug-free in vitro activation of follicles and fresh tissue autotransplantation in primary ovarian insufficiency patient: a case report and literature review. J Ovarian Res. 2018. 10.1186/s13048-018-0447-3. 30170634 10.1186/s13048-018-0447-3 PMC6119245

[45] Kawamura K, Cheng Y, Suzuki N, Deguchi M, Sato Y, Takae S, et al. Hippo signaling disruption and Akt stimulation of ovarian follicles for infertility treatment. Proc Natl Acad Sci U S A. 2013;110(43):17474–9. 10.1073/pnas.1312830110. 24082083 10.1073/pnas.1312830110 PMC3808580

[46] Sahni C, Seth S, Nayak AK, Singh R. Unraveling the mystery of multi-oocyte follicles: an observational study. Clin Ter. 2024;175(1):42–6. 10.7417/CT.2024.5032. 38358476 10.7417/CT.2024.5032

